# Multi-distribution activation energy model on slow pyrolysis of cellulose and lignin in TGA/DSC

**DOI:** 10.1016/j.heliyon.2021.e07669

**Published:** 2021-07-26

**Authors:** Jonas Kristanto, Muhammad Mufti Azis, Suryo Purwono

**Affiliations:** a Department of Chemical Engineering, Faculty of Engineering, Universitas Gadjah Mada, Yogyakarta, 55281, Indonesia; b Professional Engineering Program, Faculty of Engineering, Universitas Gadjah Mada, Yogyakarta, 55281, Indonesia

**Keywords:** Biomass pyrolysis, TGA, DSC, Kinetics, DAEM

## Abstract

Developing a kinetic model to analyze the multi-step reaction of biomass pyrolysis is pivotal to elucidate the mechanism of the pyrolysis. For this purpose, a model-fitting method such as multi-distribution the Distributed Activation Energy Model (DAEM) is one of the most reliable methods. DAEM with 4 different distribution functions of Gaussian, Logarithmic, Gumbel, and Cauchy was utilized to characterize the pyrolysis of cellulose and lignin during Thermogravimetric Analysis/Differential Scanning Calorimetry (TGA/DSC) instrumentation. By comparing Derivative Thermogravimetry (DTG) and DSC profiles, determination of pseudo-components can be done more accurately. A kinetics analysis on the pyrolysis of cellulose with a single Gaussian distribution DAEM yielded a single activation energy of 178 kJ mol^−1^ with a narrow standard deviation. This result was justified by a single and dominant endothermic peak followed by minor exothermic peaks in the DSC result. For lignin pyrolysis, the presence of multiple peaks is characterized by four pseudo-components in DAEM with activation energies of 157, 174, 194, and 200 kJ mol^−1^. These pseudo-components were confirmed by the DSC result which indicated the occurrences of two exothermic peaks with two lesser exothermic or possibly endothermic peaks at the same temperature range. These findings imply the importance of DSC to support a kinetics study of thermogravimetric pyrolysis.

## 1 Introduction

Thermal conversion of biomass is one of the main routes for biomass valorization to convert biomass to various chemicals. In general, thermochemical conversion is classified into several routes i.e. torrefaction, liquefaction, gasification, and pyrolysis with their specific purposes and products [1, 2, 3, 4]. Torrefaction is mainly used to increase the C/H ratio in solid fuel. Liquefaction, as the name suggests, focuses on the production of liquid products. Whereas gasification and pyrolysis produce various products in which the addition of oxidizing agent being the main difference in both techniques. Gasification is typically done in the presence of oxidizing agent (e.g. steam, oxygen, and carbon dioxide) while pyrolysis is conducted in the absence of any oxidizing agent.

As a part of thermochemical conversion methods, pyrolysis converts lignocellulose components to syngas under inert or N_2_ flow. As a result, pyrolysis may produce three simultaneous products: solid char, liquid oil, and pyrolytic or synthesis gas, which is usually produced around 300–600 °C [5, 6]. Numerous studies have investigated and reviewed the mechanisms, behavior, and kinetics parameters of pyrolysis [1, 7, 8]. From these studies, it is reported that hemicellulose is typically the first component to degrade, followed by cellulose and lignin [9].

Thermogravimetric Analysis (TGA) is a common instrument to study the kinetics of biomass pyrolysis. In general, the kinetics study of biomass pyrolysis on TGA focuses on the decomposition of three pseudo-components that is associated with the three main components of lignocellulosic biomass: cellulose, hemicellulose, and lignin [5, 10, 11, 12]. Each of these biomass components also have different peak thermal degradation activation energies. Cellulose decomposition activation energy is considered in the range between 175-279 kJ mol^−1^ with a low standard deviation, which indicates a uniformly occurring reaction [5, 13, 14, 15, 16]. In comparison, hemicellulose ranges between 132-186 kJ mol^−1^ with a higher standard deviation than cellulose [5, 14, 15, 16]. In contrast, lignin has the broadest range of activation energy, which ranges from 62 to 271 kJ mol^−1^ with the highest standard deviation among the three components of biomass [5, 14, 15, 16, 17, 18]. The broad range of activation energy of lignin is related to structural complexity and the variety of lignin [5, 17, 18].

The wide range of reported activation energy value of biomass pyrolysis is also due to various ways to conduct a kinetics study. For instance, in the Arrhenius Equation, by changing both the Pre-exponential factor and Activation Energy, many models can obtain the same good fit to describe the TGA result of biomass pyrolysis. This occurrence is usually called a compensation effect, which enables different sets of preexponential factors and activation energies resulting in the same reaction profile [10, 19, 20, 21]. One alternative route to achieve a more consistent result is by applying multiple constant preexponential factors to obtain sets of activation energy values [20, 22, 23]. However, this may cause redundancy and difficulty to replicate. Another approach is to fix the value of the preexponential factor for each component or pseudo-components with reasonable values such as based on transition-state theory [10].

To obtain kinetic parameters of the reaction, various methods can be applied. Usually, the kinetics study is divided into 2 categories: model-free and model-fitting method. Model-free or iso-conversional such as the Kissinger-Akahira-Sunose, Flynn-Wall-Ozawa, and Coats-Redfern methods is usually easier to be applied since it only requires contemporary linear regression which does not require a high computational cost [24, 25, 26]. However, most of the model-free methods usually needs a minimum of 3 experiments at different heating rates to be applied accordingly [27]. Additionally, the result from a kinetics study with a model-free method is not suitable to identify multi-step reactions [27, 28].

In complex cases such as biomass pyrolysis, which consists of multiple reaction steps, a model-fitting method is more suitable to be applied [27]. One of the most popular and widely used models is the Distribution Activation Energy Model (DAEM). DAEM assumes a series of first-order-irreversible-parallel reactions to explain multiple reactions that take place in the pyrolysis process [13, 29, 30]. Due to the complexity and tremendous diversity in biomass, various approaches to use DAEM have been implemented on isolated biomass components which have resulted in more detailed kinetics parameters [6, 15, 17, 18]. This application leads to a better understanding of the macroscopic parameters of each biomass pyrolysis mechanism.

Determining the number of pseudo-components on a macroscopic scale is useful since these three components have distinct pyrolysis behavior. Nevertheless, this approach tends to simplify the pyrolysis sequence's proper mechanistic behavior by depleting minor reactions with a mathematic generalization. The use of pseudo-components has also been developed for 4 to 5-pseudo-components to describe the decomposition of plastic waste, lignocellulosic, and marine biomass [31, 32, 33, 34]. Two studies reported a detailed approach with more pseudo-components for cellulose, hemicellulose, and lignin [6, 15]. However, the number of pseudo-components used was still mainly based on a mathematical fit with a limited description of the mechanistic behavior. Determination of pseudo components is likely related to the reaction enthalpy profile. However, the relation between kinetics parameters and the nature of each reactions' parameters, such as enthalpy, has not been widely explored, especially to determine the number of pseudo-components in the DAEM kinetics model. By using a Differential Scanning Calorimetry (DSC) instrument, thermal behavior such as enthalpy profiles of each reaction step can be recorded based on the supplied energy from the instrumentation. Hence, DSC provided the thermodynamics properties of the reactions.

The objective of the present work is to develop a kinetic model based on DAEM to describe cellulose and lignin decomposition using 4 distribution models namely Gaussian, Logistic, Gumbel, and Cauchy distribution. Here, TGA/DSC instrumentation was used as a pyrolysis chamber of both components under an inert N_2_ atmosphere. Commercial cellulose and lignin were chosen to represent the two main biomass components as well as to ensure the reproducibility of this work. Four different distribution functions and a first-order DAEM with constant preexponential factor value (1.67 × 10^13^ s^−1^) were utilized to obtain the kinetics parameters and the number of pseudo-components in the pyrolysis of lignin and cellulose. Additionally, the correlation between the Derivative Thermogravimetry (DTG) and DSC profiles of each sample was utilized to confirm the number as well as the thermodynamic properties of each pseudo-components.

## 2 Materials and methods

### 2.1 Materials

Commercial cellulose of avicel PH-101 (11365) was used as received to represent cellulose. Alkali lignin with low sulfonate content (Sigma Aldrich, 471003) was also used for this work. Each sample was pelletized (ø 4 mm; 1 mm in height cylindrical shapes) prior to the pyrolysis experiment.

### 2.2 Pyrolysis

The pyrolysis experiment was conducted in a TGA/DSC PT 1000 Linseis under an inert atmosphere (at 50 mL min^−1^ of 99.99% nitrogen gas) with a constant heating rate of 5 °C min^−1^ 10–20 mg of samples were pyrolyzed from 22 °C to 600 °C. The low heating rate was selected to minimize the thermal lag (thermal hysteresis) between thermocouple reading and exact sample temperature [5]. Each experiment was conducted twice, and both data were used in the modeling.

### 2.3 Data filtering

To remove noise from received TGA and DSC data, the Savitzky-Golay data filtering technique was utilized. The Savitzky-Golay technique involves a polynomial smoothing method to small portion of data and has been widely utilized in many thermogravimetric works [35, 36, 37]. This technique was implemented to give a better representation of the data without altering the true nature of the readings from the instrument.

### 2.4 Pyrolysis kinetics modeling

DAEM was implemented in this work to explain the pyrolysis of each sample. DAEM contains multiple parameters such as activation energies (E) and preexponential factors ($k_{0}$) to explain the irreversible parallel reactions which occurs according to the distribution function as presented in Eq. (1).

(1) $$\frac{dx}{dT}=\int_{0}^{\infty }\left\{\frac{k_{0}}{\beta }\exp\left[-\left(\frac{E}{RT}+\frac{k_{0}}{\beta }\overset{T}{\underset{T_{0}}{\int }}\exp\left(-\frac{E}{RT}\right)dT\right)\right]\right\}F\left(E\right)dE$$

Here, R is the ideal gas constant, T is temperature, β is the heating rate, and x is the mass-based conversion degree as described in Eq. (2).

(2) $$x=\frac{m_{0}-m}{m_{0}-m_{\infty }}$$

whereas $m_{0}$ is the initial mass of the sample, $m_{\infty }$ is the remaining mass of the sample at the end of the experiment, and m is the temporal measured mass. In addition, F(E) describes the distribution model of activation energy and here we have proposed to use 4 types of distribution models namely Gaussian, Logistic, Gumbel, and Cauchy. The Gaussian function is described by (3).

(3) $$F{\left(E\right)}_{i}=\frac{1}{\sigma_{i}\sqrt{2\pi }}\exp\left[-\frac{1}{2}{\left(\frac{E-{\bar{E}}_{i}}{\sigma_{i}}\right)}^{2}\right]$$

In addition, we have also proposed the use of the following Logistic (4), Gumbel (5), and Cauchy function (6) as follows:

(4) $$F{\left(E\right)}_{i}=\frac{1}{4\sigma_{i}}\text{sech}\left(\frac{E-{\bar{E}}_{i}}{2\sigma_{i}}\right)$$

(5) $$F{\left(E\right)}_{i}=\frac{1}{\sigma_{i}}\exp\left[-\left(\frac{E-{\bar{E}}_{i}}{\sigma_{i}}+\exp\left\{-\left(\frac{E-{\bar{E}}_{i}}{\sigma_{i}}\right)\right\}\right)\right]$$

(6) $$F{\left(E\right)}_{i}=\frac{1}{\pi \sigma_{i}}{\left[1+{\left(\frac{E-{\bar{E}}_{i}}{\sigma_{i}}\right)}^{2}\right]}^{-1}$$

**Figure 1:**
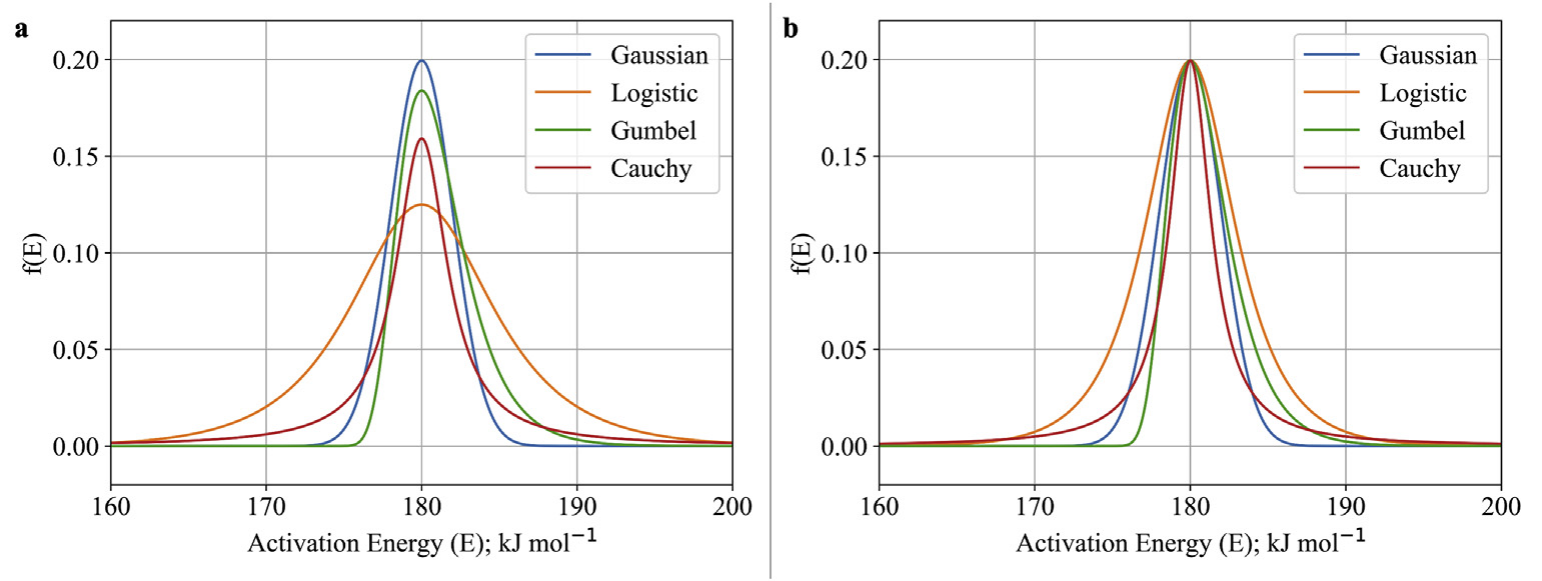
Comparison of distribution function at the same a) parameter value and b) peak value.

The comparison of these 4 distributions is presented in Figure 1. As seen in Figure 1a, the use of the same parameters of ${\bar{E}}_{i}$ and $\sigma_{i}$ generate different behavior. Here, the value of ${\bar{E}}_{i}$ and $\sigma_{i}$ were set to 180 kJ mol^−1^and 2 kJ mol^−1^, respectively. In general, it could be inferred that the Logistic distribution generated the lowest peak distribution value. However, the Logistic distribution also provides the highest variance of activation energy. On the other hand, the Gaussian distribution resulted in the highest peak value among the 4 provided distributions. Whereas the Cauchy and Gumbel distribution tend to have moderate peaks and small variances which lies between the Logistic and Gaussian distributions. Additionally, a sensitivity test of these distributions was also made to generate the same peak as represented in Figure 1b. Here, the value of $\sigma_{i}$ was manipulated to obtain the same peak. As seen in Figure 1b, the Logistic distribution gave a larger area of the chart than the other three distributions. Hence, it infers that each distribution function has specific characteristics for later use in adapting DAEM on biomass pyrolysis. Identification of pseudo-components was made by introducing a multi-distribution factor to represent each pseudo-component ($c_{i}$) which totals to the amount of dx/dT.

(7) $$\frac{dx}{dT}=\overset{N}{\underset{i=1}{\sum }}\left(c_{i}{\frac{dx}{dT}|}_{i}\right)$$

Each pseudo-component has its contribution fractions ($c_{i}$) which sums up to 1. In addition, each component also possesses a certain preexponential factor ($k_{0}$), activation energy ($\bar{E}$), and standard deviation (σ). To obtain these parameters, Eq. (1) through (7) were numerically solved in Spyder (Python IDE). This method is a refinement method from the previous work of Güneş and Güneş [38].

The objective function (8) was used to estimate parameters in DAEM by taking residuals from subtracting the dx/dT from the experimental data and simulation. The value generated by Eq. (8) was minimized in Spyder (Python IDE) by using the *scipy.optimize.least_square* module to minimize the residual between simulated and experimental data.

(8) $$residual=\left({dx/dT|}_{exp}-{dx/dT|}_{sim}\right)$$

The non-linear regression was used to estimate 3 parameters: the contribution fraction ($c_{i}$), activation energy ($\bar{E}$), and standard deviation (σ) while the preexponential factor ($k_{0}$) was set at a constant value of 1.67 × 10^13^ s^−1^. For comparison, we also calculated the coefficient of determination (R^2^) for each result.

## 3 Results and discussion

### 3.1 Thermogravimetry result of cellulose and lignin

**Figure 2:**
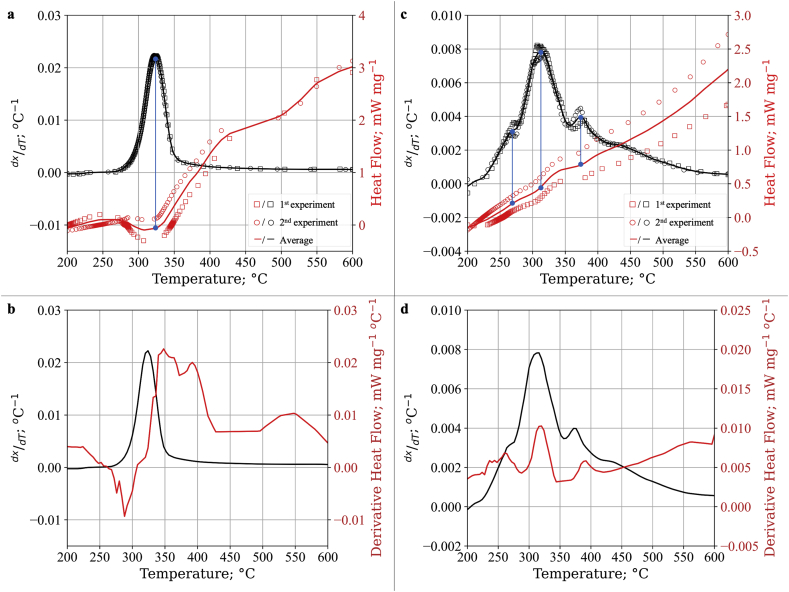
Thermogravimetric curve of a,b) cellulose and c,d) lignin at 5 °C min-1 heating rate from 200 to 600 °C.

Figure 2 shows the results from TGA experiments describing the thermal decomposition and heat flow of cellulose (panel a and b, respectively) as well as lignin (panel c and d, respectively). Both data from 2 experiments were denoted by squares and circles, respectively. These two experiments showed consistent results as shown by a solid line indicating the average values between each experiment. It is important to note that the data analysis was conducted from 200 °C, as moisture removal and evaporation of light volatiles mainly occurs below this temperature [7]. This behavior also occurs during the pyrolysis of cellulose or lignin. Thus, these events were considered as the pre-pyrolysis stage.

Figure 2a shows that the thermal decomposition of cellulose which gives a single distinct peak DTG at ca. 330 °C. As the temperature increased, cellulose starts to degrade substantially at ca. 270 °C. Subsequently, the degradation of cellulose continuously increases reaching a peak at 330 °C. The same single DTG peak illustrated in Figure 2a is consistent with the thermogravimetric pyrolysis study of a similar type of cellulose as reported in literatures [9, 20]. However, the peak was located slightly differently due to thermal hysteresis as a result of different heating rates (5 vs 10 °C min^−1^).

The DSC result shown in Figure 2a (solid red line) indicated a negative value which indicates the occurrence of an endothermic process at 330 °C. This occurrence is precisely located at the same temperature range as the maximum mass loss shown by the DTG result in Figure 2a. Furthermore, DTG and DSC showed different results at higher temperatures. DTG showed a lack of any distinct pattern of reaction whereas DSC showed the occurrence of multiple changes of the supplied heat flow. However, these fluctuations of the DSC result were considered minor as the DTG result did not show any significant or distinct pattern, thus indicating a lack of significance in this temperature range.

Figure 2c shows the thermal decomposition of lignin. As seen here, the lignin sample gave relatively slow decomposition during the experiment. In contrast to cellulose, the DTG profile of lignin clearly shows the presence of multiple overlapping peaks at 270 °C, 310 °C, and 380 °C. This finding differs slightly from a previous thermogravimetric pyrolysis study on the same type of lignin where only one distinct peak was displayed at around 320 °C [15]. However, other studies on various types of lignin using a lower heating rate showed the same profile as reported in this work [17, 39, 40]. This highlights the importance of the use of a lower heating rate for a thermogravimetric study on lignin pyrolysis to avoid significant thermal hysteresis which may poorly resolve overlapping DTG peaks.

The result from the DTG curve in Figure 2c was also confirmed by the DSC profile (Figure 2c). From the DSC result, lignin undergoes continuous exothermic decomposition from 250 °C to the final temperature. Fluctuation of DSC results also occurred around 270 °C through 375 °C, indicating the different phases of lignin pyrolysis reactions with various enthalpies of reactions. This phenomenon was also highlighted further in the derivative form of DSC (solid red line in Figure 2d). Thereby, three overlapping DTG peaks at 270 °C, 310 °C, and 375 °C, followed by char decomposition may be considered as four different stages of lignin pyrolysis.

### 3.2 Kinetics of cellulose and lignin thermogravimetric pyrolysis

**Figure 3:**
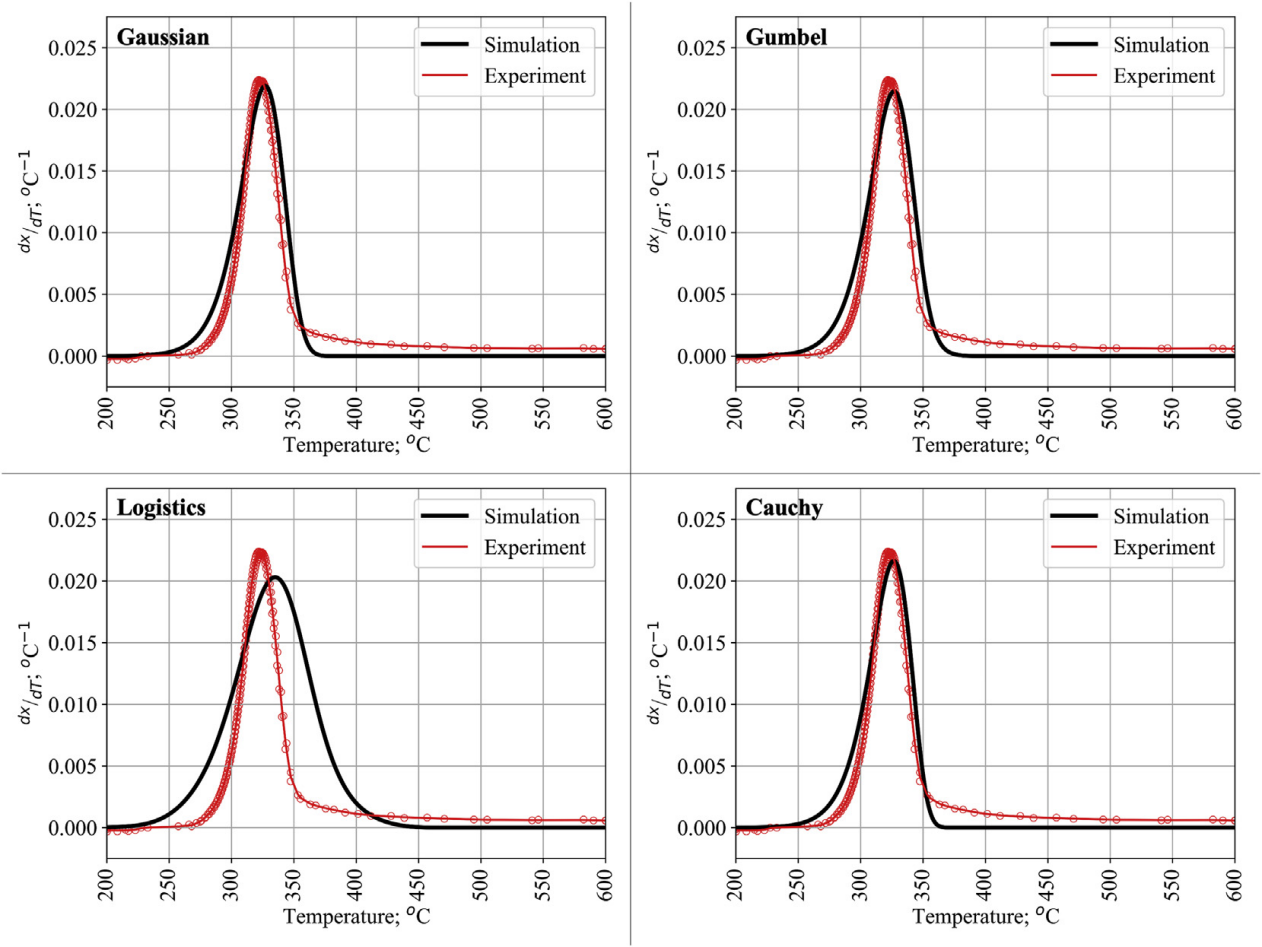
Experimental and calculated DTG curve of cellulose.

In general, the implementation of DAEM to describe the DTG curve of cellulose and lignin requires a few specific considerations such as the suitability between the shape and number of peaks shown in DTG to the applied distribution function. From Figure 2a, DTG and DSC results of cellulose indicated that the use of one prominent reaction peak is sufficient to represent the pyrolysis of cellulose. Figure 3 shows the modeling result of DAEM to the DTG curve for cellulose using the four distribution models. The use of a single pseudo-component in cellulose pyrolysis already satisfies the experimental data with the coefficient of determination (R^2^) around 0.9305 for the Gaussian distribution. As this result suggest, cellulose pyrolysis is practically dominated by a thermal decomposition around 270–350 °C. The calculated Gaussian distribution average activation energy of cellulose decomposition was 178.6488 kJ mol^−1^. This value of activation energy and its low standard deviation of activation energy is in good agreement with previous work which reported an activation energy within the ranges of 175–279 kJ mol^−1^ [5,13, 14, 15, 16].

Among the four applied distribution functions, the Logistic distribution is considered as the worst as depicted in Figure 3 and compared in Table 1. This finding contradicts Fiori et al. [19], who suggested that the Logistic distribution was fitted slightly better than the Gaussian distribution for grape remnant pyrolysis and Cai et al. [41] with an unidentified cellulose. However, direct comparison of those reports with our work is vague as the different cellulose is used [41] and the pseudo-component approach was not utilized in the [19]. In comparison, the Cauchy distribution provided a better fit to cellulose pyrolysis (Table 1). However, the advantage of using the Cauchy distribution is not tremendous compared to the Gaussian and Gumbel distributions.

**Table 1:** Kinetics parameters of cellulose pyrolysis.

Parameter	Gaussian	Logistic	Gumbel	Cauchy
$E_{0}$; kJ mol^−1^	178.6488	182.2636	177.8279	178.2789
σ; kJ mol^−1^	1.6320	2.7686	1.6634	0.0052
R^2^	0.9305	0.7417	0.9338	0.9486

From Figure 2c, DTG profile of lignin consists of one dominant peak at 310 °C two overlapping minor peaks at 270 and 375 °C, followed by char decomposition above 375 °C. Compared to Figure 2a, the char decomposition peak at 425 °C in lignin pyrolysis has a larger contribution to mass loss than that for cellulose pyrolysis. Thereby, it is important to involve the char decomposition peak to describe lignin pyrolysis.

**Figure 4:**
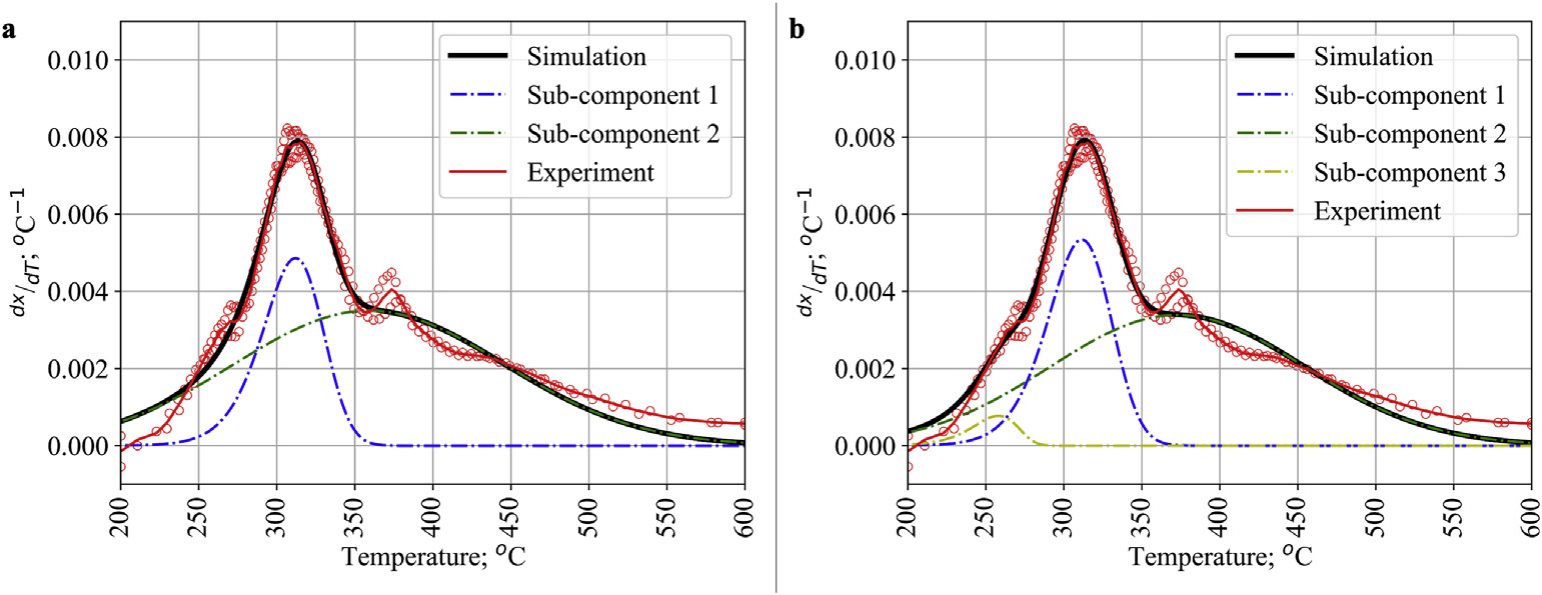
Experimental and calculated DTG curve of lignin with a) 2 pseudo-components and b) 3 pseudo-components Gaussian DAEM.

In Figure 4a, we have tested the use of two pseudo-components using Gaussian DAEM to describe the DTG curve of lignin pyrolysis. From our calculation, the model gave an R^2^ value of 0.9864 with an activation energy around 174.5420 kJ mol^−1^ and 190.9414 kJ mol^−1^ for the first and second pseudo-components, respectively. This value is consistent with other literature with the same methodology and subject which resulted in activation energy values around 176.64 kJ mol^−1^ and 188.94 kJ mol^−1^ for the first and second pseudo-component, respectively [15]. However, the simulation did not capture well the peak at 350–400 °C as well as at the beginning of the pyrolysis sequence. This finding has compelled us to investigate the application of a three pseudo-components Gaussian DAEM.

The DTG curve of lignin pyrolysis was subjected to three pseudo-components with the Gaussian DAEM as illustrated in Figure 4b. The activation energies resulting from this fitting were 174.5508 kJ mol^−1^, 195.1852 kJ mol^−1^, and 157.3830 kJ mol^−1^. Different from Figure 4a with 2 pseudo components, the current fitting with 3 pseudo-components showed a better fit at lower temperature or at the initial step of lignin pyrolysis which resulted in a higher R^2^ value of 0.9882. Nevertheless, the model still cannot capture the experimental data at higher temperatures which were precisely visualized at around 370 °C in Figure 4b. Therefore, it is envisaged to add one more distribution to represent the fourth pseudo-component.

**Figure 5:**
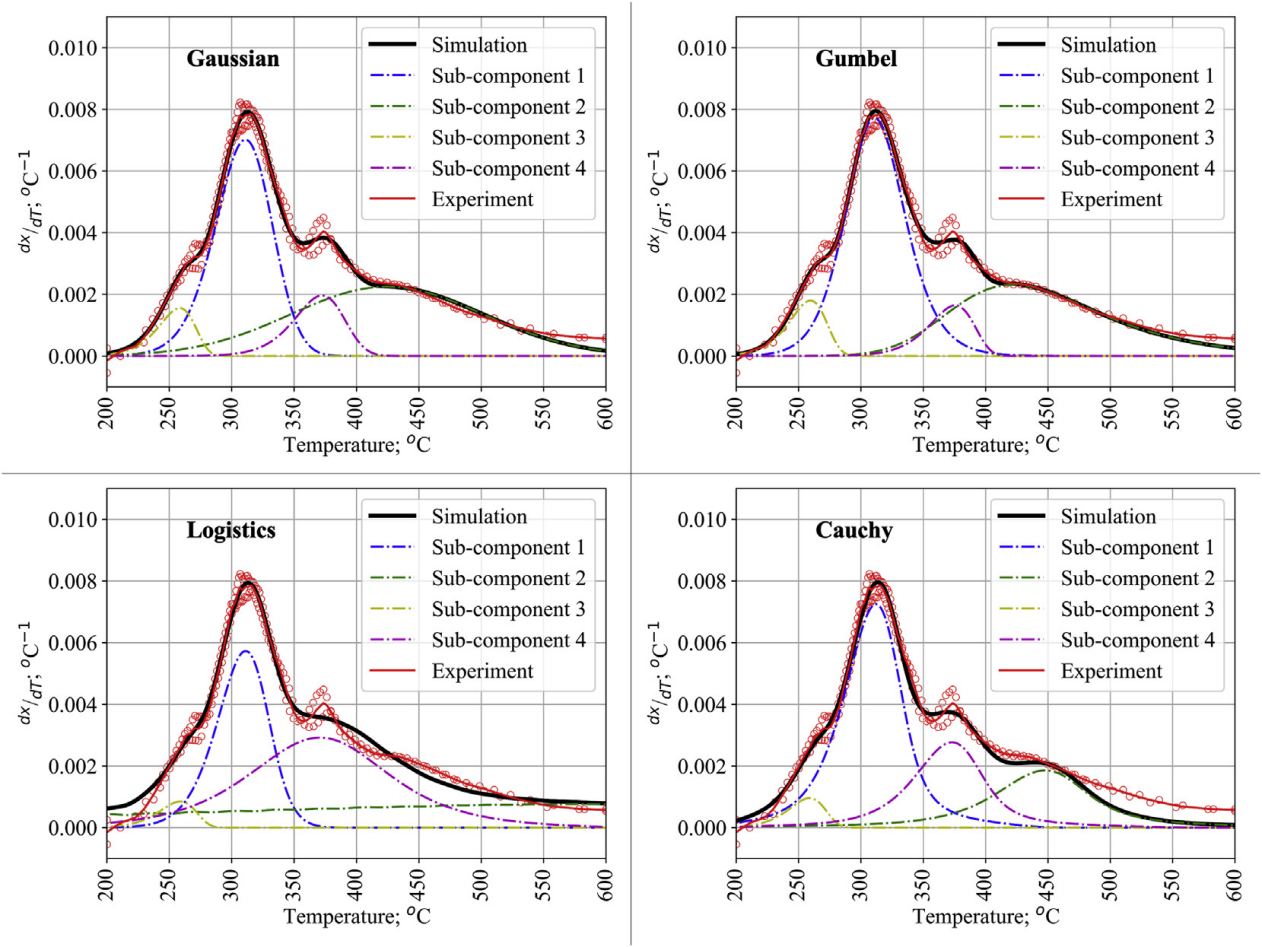
Experimental and calculated DTG curve of lignin with 4 pseudo-components DAEM Comparison of DTG and DSC value on the pyrolysis of a) cellulose and b) lignin.

Figure 5 shows the result of four distributions in DAEM to simulate the DTG curve of lignin pyrolysis. Our modeling result shows that the use of four pseudo-components fitted well to the lignin pyrolysis sequence with an R^2^ value of 0.9936 for the Gaussian distribution, which is higher than the previous DAEM application in Figure 4. Between each distribution function model, Gaussian and Gumbel distributions provided higher R^2^ values for lignin pyrolysis rather than the Cauchy and Logistic distributions as compared in Table 2 and visually represented in Figure 5. From Figure 5, the Logistic distribution had an advantage with a broader range reaction, specifically the second pseudo-component reaction, which agrees with a previous finding [42]. This was also confirmed by the behavior of the Logistic distribution at the same peak value shown in Figure 1b. In contrast, the Cauchy distribution fits better with a swiftly occurring reaction. At the same time, the Gaussian and Gumbel distributions provide a middle ground between each extreme.

**Table 2:** Kinetics parameters of lignin pyrolysis.

Model	Parameter	Pseudo-component			
		1^st^	2^nd^	3^rd^	4^th^
Gaussian	$c_{i}$	0.4070	0.4312	0.0599	0.1019
	$E_{0}$; kJ mol^−1^	174.6626	211.1543	157.4708	192.7969
	σ; kJ mol^−1^	4.6001	22.6944	0.0126	2.6252
	R^2^	0.9936			
Logistic	$c_{i}$	0.1967	0.4999	0.0211	0.2823
	$E_{0}$; kJ mol^−1^	174.3412	265.3994	157.6159	194.1333
	σ; kJ mol^−1^	1.3342	50.6039	0.0392	6.8136
	R^2^	0.9901			
Gumbel	$c_{i}$	0.4832	0.3696	0.0705	0.0767
	$E_{0}$; kJ mol^−1^	173.5402	209.1221	157.5433	193.2413
	σ; kJ mol^−1^	4.8764	17.0193	0.6483	0.0002
	R^2^	0.9937			
Cauchy	$c_{i}$	0.5021	0.2235	0.0396	0.2348
	$E_{0}$; kJ mol^−1^	174.6224	216.9202	157.6159	193.4639
	σ; kJ mol^−1^	3.5543	8.6365	0.0023	4.9539
	R^2^	0.9897			

The number of pseudo-components is likely associated with the number of distinct reactions. Zhang et al. already applied two pseudo-components of lignin in their pyrolysis stage (100–550 °C) with the same low sulfonate content alkali lignin [15]. Meanwhile, we applied 4 pseudo-components DAEM for lignin pyrolysis from 200 °C to 600 °C suggesting the occurrence of 4 distinct reactions.

Table 2 shows the kinetic parameters obtained from lignin pyrolysis. The larger standard deviation of activation energy with lignin pyrolysis compared to that of cellulose suggests the occurrence of a concatenation of reactions during lignin pyrolysis. However, two smaller and minor peaks presented as third and fourth pseudo-components have a lesser standard deviation and smaller fraction than the first and third pseudo-component peaks. This indicates two swift minor reactions that may not have been recorded before in the previous kinetic study [15]. These two reactions add up to 14.44% of the total lignin pyrolysis between 200-600 °C may seem minor and negligible at first and are discussed in the following section.

### 3.3 Correlation between kinetics study and DSC result

**Figure 6:**
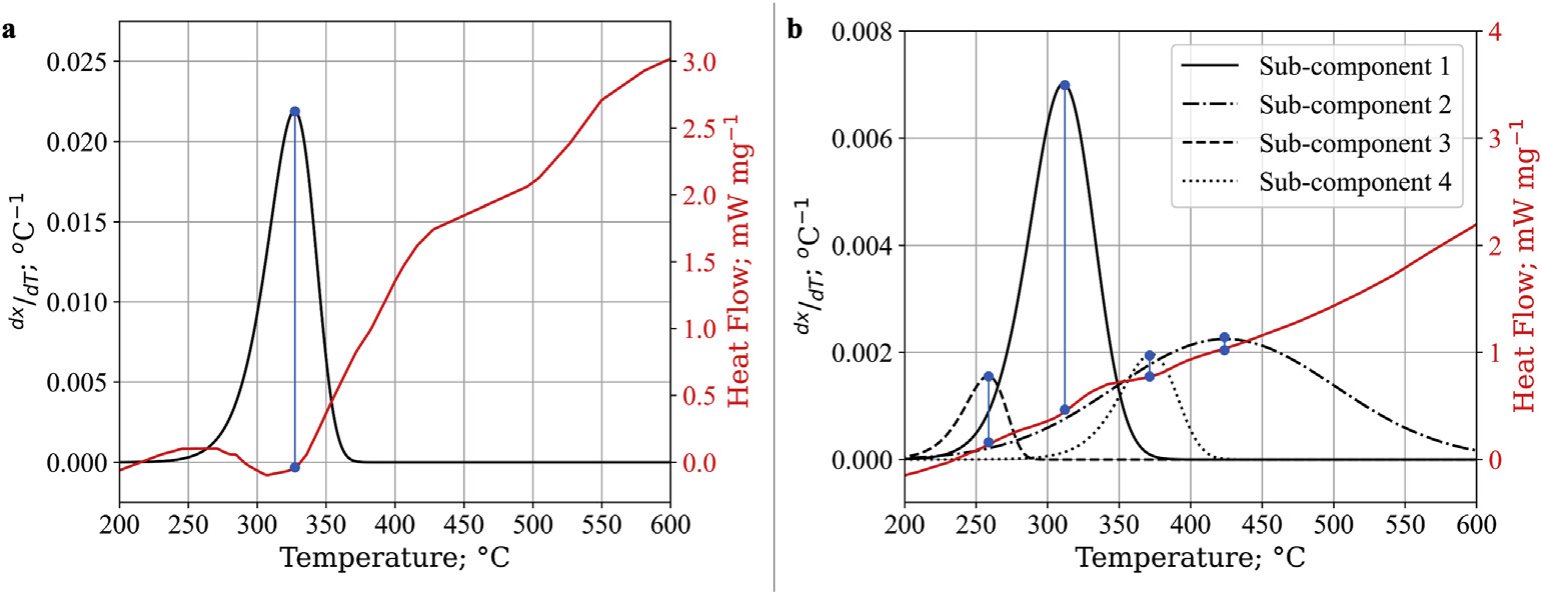
Comparison of DTG and DSC value on the pyrolysis of a) cellulose and b) lignin.

As described earlier, we have also investigated the correlation between the kinetics study and the DSC results. The use of the DSC result enabled us to predict the number of pseudo-components in cellulose and lignin thermogravimetric pyrolysis. To our knowledge, there is still limited work in the literature that correlates the kinetics study of biomass pyrolysis to DSC results in determining the number of pseudo-components [5, 12, 31, 32, 33, 34, 43, 44, 45, 46, 47]. It is also important to note that the use of aggressive data filtering and a higher heating rate may conceal minor DTG peaks. In this study, one pseudo-components and four pseudo-components were respectively used in cellulose and lignin pyrolysis kinetics. The number of used pseudo-components is also rationalized by comparing DTG and DSC curves, as seen in Figure 6 and discussed before in section 3.1.

From the development of the kinetics model on pyrolysis, a set of kinetics parameters containing activation energy, standard deviation, and fraction were yielded. These results were compared to DSC results to describe the thermodynamic properties of each reaction as summarized in Table 3. In Figure 6a, a reverse DSC peak around 325 °C is within the range of the DTG peak around the same temperature. The negative value of this peak indicates cellulose undergoes endothermic pyrolysis. At temperatures below 290 °C and above 330 °C, cellulose undergoes exothermic reactions with DSC heat flow fluctuations from 430-550 °C. These exothermic reaction sequences and fluctuation are not displayed in the DTG result. Moreover, as previously discussed, a single pseudo-component in cellulose pyrolysis kinetics modeling already gave a satisfying and practical solution. Therefore, a single endothermic reaction is practical and adequate to describe the cellulose pyrolysis sequence.

**Table 3:** Kinetics parameters on cellulose and lignin pyrolysis and its thermodynamics properties.

Sample		Kinetics Parameter			Maximum Mass Loss Temperature; °C	Thermodynamic Property
		$c_{i}$	$E_{0}$; kJ mol^−1^	σ; kJ mol^−1^		
Cellulose		1	178.6488	1.6320	330	Endothermic
Lignin						
pseudo-component	1^st^	0.4070	174.6626	4.6001	310	Possibly endothermic
	2^nd^	0.4312	211.1543	22.6944	425	Exothermic
	3^rd^	0.0599	157.4708	0.0126	260	Exothermic
	4^th^	0.1019	192.7969	2.6252	372	Possibly endothermic

Although the DSC reading on lignin pyrolysis continuously shows persistent exothermic reactions, a few fluctuations were recorded in Figure 6b and previously highlighted in Figure 2d. Continuous positive heat flow corresponds to the second pseudo-component reaction, which occurs in the widest span of the temperature range. A slight increase in heat flow around 250 °C indicates an exothermic reaction of the third pseudo-component. The decrease in heat flow around 270 °C–310 °C and between 340 °C to 375 °C indicated a lesser exothermic or possibly endothermic reactions from the pyrolysis of the first and fourth pseudo-component. This result may have not been achieved if DSC were not used since the second and fourth pseudo-component peak might be considered minor and possibly filtered out from data analysis. In the end, considering the presence of multistage reaction in lignin pyrolysis, combination of TGA and DSC data resulted in more reliable and precise justifications in determining the number of pseudo-components.

## 4 Conclusions

Cellulose and lignin thermogravimetric pyrolysis have been investigated by using multi distribution DAEM coupled with 4 types of distribution functions. Combining the use of DTG and DSC results may serve as the basis to determine the number of pseudo-components used in kinetic modeling supported by the difference in thermodynamic properties. Cellulose decomposition showed a single distinct peak with $\bar{E}$ around 178 kJ mol^−1^. Whereas lignin pyrolysis showed the presence of 4 pseudo-components with $\bar{E}$ 157, 174, 194, and 200 kJ mol^−1^ respectively. Therefore, the method demonstrated in this work may increase our understanding of the kinetics analysis of thermogravimetric pyrolysis. Furthermore, the result from this research may also serve as a reference for future research on elucidating the mechanism of thermogravimetric pyrolysis by using a combined kinetic study with DSC profiles. For further development, it also opens a pathway to conduct a more thorough kinetics study coupled with thermodynamics modeling of the DSC profile.

## Declarations

### Author contribution statement

Jonas Kristanto: Conceived and designed the experiments; Performed the experiments; Analyzed and interpreted the data; Wrote the paper.

Muhammad Mufti Azis: Conceived and designed the experiments; Contributed reagents, materials, analysis tools or data; Wrote the paper.

Suryo Purwono: Contributed reagents, materials, analysis tools or data; Wrote the paper.

### Funding statement

This work was supported by the 10.13039/501100005981Direktorat Jenderal Pendidikan Tinggi (ID) (38/E1/KPT/2020).

### Data availability statement

Data will be made available on request.

### Declaration of interests statement

The authors declare no conflict of interest.

### Additional information

No additional information is available for this paper.
